# Understanding plant reproductive diversity

**DOI:** 10.1098/rstb.2009.0199

**Published:** 2010-01-12

**Authors:** Spencer C. H. Barrett

**Affiliations:** Department of Ecology and Evolutionary Biology, University of Toronto, 25 Willcocks Street, Toronto, Ontario M5S 3B2, Canada

**Keywords:** dioecy, floral diversity, plant sex, mating, self-fertilization, wind pollination

## Abstract

Flowering plants display spectacular floral diversity and a bewildering array of reproductive adaptations that promote mating, particularly outbreeding. A striking feature of this diversity is that related species often differ in pollination and mating systems, and intraspecific variation in sexual traits is not unusual, especially among herbaceous plants. This variation provides opportunities for evolutionary biologists to link micro-evolutionary processes to the macro-evolutionary patterns that are evident within lineages. Here, I provide some personal reflections on recent progress in our understanding of the ecology and evolution of plant reproductive diversity. I begin with a brief historical sketch of the major developments in this field and then focus on three of the most significant evolutionary transitions in the reproductive biology of flowering plants: the pathway from outcrossing to predominant self-fertilization, the origin of separate sexes (females and males) from hermaphroditism and the shift from animal pollination to wind pollination. For each evolutionary transition, I consider what we have discovered and some of the problems that still remain unsolved. I conclude by discussing how new approaches might influence future research in plant reproductive biology.

## 1. Introduction

The astonishing reproductive diversity of flowering plants has attracted the senses and curiosity of humans since the dawn of civilization. Flowers have long played a prominent role in religious ceremonies and are the subject of countless artistic endeavours. Through breeding and artificial selection, plants provide the food supply that forms the basis of human civilization, and through horticulture the ornamentals that adorn our cities and gardens. Understanding plant reproduction is of immense practical importance for biotechnology, the conservation of biodiversity and the control of invasive species. In this short essay, I hope to demonstrate why plant sexual diversity also presents many intriguing challenges and opportunities for the study of evolution and adaptation.

Why should the reproductive structures of flowering plants (angiosperms) exhibit greater variety than those of any other group of organisms? This question is particularly perplexing when one considers that they serve just one main function—to promote mating. The answer lies in the immobility of plants and their need to engage the services of pollen vectors to ensure cross-pollination and the production of offspring of high genetic quality. Rather than rely on wind, or a single group of animal pollinators, the reproductive structures of the majority of flowering plants have undergone spectacular waves of diversification depending on the local abundance and efficacy of available pollen vectors. Pollinator-driven speciation has played an important role in the evolution of angiosperm diversity.

Diversification in form and function of flowers is associated with an equally impressive variety of mating strategies and sexual systems. The fundamental hermaphroditic condition of most angiosperm species facilitates opportunities for both cross- and self-fertilization, and because of the modular growth of plants and production of numerous flowers, multiple paternity is commonplace. Patterns of mating in plants can be both complex and highly promiscuous in comparison with many animal groups. Male and female gametes are deployed in a wide array of spatial and temporal options at the flower, inflorescence, plant and population level resulting in diverse sexual systems composed of different combinations of hermaphroditic, female and male plants. Understanding the causes and mating consequences of this sexual diversity has been an enduring source of curiosity since the birth of the biological sciences.

Carl Linnaeus used variation in sexual structures as the basis of his plant classification, Charles Darwin conducted breeding experiments on plants and wrote three important books on the different facets of plant reproduction, and Ronald Fisher developed the population genetic principles that form the basis of the modern analysis of plant mating-system evolution. Today, research on plant reproductive biology is one of the most dynamic and popular fields in ecology and evolutionary biology. This appeal may arise from the integrated nature of research that begins with field observations of natural history, but also involves theory, comparative biology, genetics, ecology and in many cases the study of plant–animal interactions. Early work on plant reproduction tended to concentrate on species from temperate ecosystems, but today studies of tropical ecosystems are routinely providing new insights on floral evolution and adaptation.

Here, I provide some personal reflections on several main research themes concerned with plant reproductive diversity, paying particular attention to work on the evolution of pollination and mating strategies. I begin by sketching a brief history of milestones in this field starting with early studies by naturalists, through theory development and experimental tests, to contemporary investigations using molecular tools. The main focus of my review concerns three major angiosperm evolutionary transitions—the pathway from outcrossing to predominant selfing, the origin of the separate-sexed condition (dioecy) from hermaphroditism and the shift from animal pollination to wind pollination. I chose these topics because of my own interest in them, and the order in which they are discussed corresponds roughly to a gradient in the depth of the general understanding of each transition. We know quite a lot about the evolution of selfing, a fair bit about why dioecy arises and relatively little about the evolution of wind pollination. These evolutionary transformations in pollination and mating systems provide me with opportunities to illustrate several general principles but also to highlight problems that need resolving. I conclude by briefly considering how plant reproductive biology may evolve over the next few decades through the integration of new techniques and in response to the challenges presented by global environmental change.

## 2. A brief historical sketch

A practical understanding of plant sexuality was evident approximately 2000 BP when Egyptians crossed male and female plants of the date palm to produce fruits. Studies on flower pollination began much later in the seventeenth and eighteenth century when the early naturalists, notably Christian Konrad Sprengel, Joseph Kölreuter and Thomas Knight, began to interpret floral function and make controlled cross- and self-pollinations (reviewed in Baker 1979). This work was the prelude to Charles Darwin's observations and experiments at Down House, synthesized in his three books on the different aspects of plant reproduction (Darwin 1862, 1876, 1877). In these works, Darwin provided the conceptual foundation for future research on the evolution and adaptive significance of variation in pollination and mating systems and many of his ideas remain remarkably durable today (reviewed in Barrett 2010).

During the 1930–1950s, the neo-Darwinian synthesis stimulated work on the genetics of plant reproductive systems. Both Ronald A. Fisher and J. B. S. Haldane investigated the inheritance of sexual morphs in heterostylous plants, and Fisher's (1941) incisive analysis of the conditions influencing the spread of a gene causing self-fertilization was the first application of population genetic principles to mating-system evolution. Subsequent growth in biosystematics led to increasing awareness that reproductive systems play a central role in governing the patterns of variation within and among plant populations, with important taxonomic consequences. Unfortunately, influential work on the evolution of genetic systems during this period was based on the notion that the amount of genetic variation in a population was the main target of selection, with inbreeding and outbreeding promoting ‘immediate fitness’ versus ‘long-term flexibility’, respectively (Darlington 1939). However, by the 1970s, the difficulty with this essentially ‘group-selection’ perspective was exposed largely through the work of David Lloyd (reviewed in Barrett & Charlesworth 2007), and a return to models of individual selection in the Fisherian tradition followed.

Up to this time, inferences about mating patterns in plant populations were largely based on observations of flower morphology and the patterns of phenotypic variation within populations. A major breakthrough came with the use of allozyme polymorphisms for directly measuring mating parameters in populations based on multiple loci (Brown & Allard 1970). Estimates of outcrossing rates, inbreeding coefficients and paternity in diverse species rapidly accumulated and today genetic markers are integral to the study of plant mating. As data accumulated, one of the key findings was that the distribution of outcrossing rate (*t*), or its complement the selfing rate (*t* = 1−*s*), among species of animal-pollinated plants was bimodal, with an excess of predominantly outcrossing or highly selfing species (Schemske & Lande 1985). Theoretical models based on the joint evolution of selfing and inbreeding depression predicted this general pattern (Lande & Schemske 1985), leading to considerable experimental work on the relation between selfing rates and the intensity and life-history dynamics of inbreeding depression (Husband & Schemske 1996). However, doubts have recently been raised about the adequacy of the existing comparative data for testing theories that predict bimodality (Igic & Kohn 2006). Recent attention has now focused on the selective forces that might maintain both selfing and outcrossing, and whether this ‘mixed mating’ represents a stable evolutionary strategy (Goodwillie *et al*. 2005; Harder *et al*. 2008), or is simply a non-adaptive cost of geitonogamous (between flower) self-pollination resulting from the display of many flowers simultaneously and local foraging by pollinators (Harder & Barrett 1995).

Despite Fisher's fundamental insights on the evolution of selfing, most work in plant reproductive biology until the 1970s was descriptive and largely non-process orientated. However, around this time a theoretical framework began to develop through the work of David Lloyd, Deborah and Brian Charlesworth and Eric Charnov. Their application of population genetic and phenotypic selection models for understanding the evolution of mating strategies led to what has been described as the ‘new plant reproductive biology’ in which fertility, gender equality, marginal values and genetic accounting emerged as central theoretical concepts (Morgan & Schoen 1997). These developments, in concert with the growth of evolutionary ecology as a vibrant discipline, resulted in a rejuvenation of the field and led to stronger and more focused research in which hypothesis testing of theory through field experiments characterized the best research.

The spatial and temporal scales in which plant reproductive diversity is now investigated have broadened substantially. Earlier work was often rather myopic in scope, involving species-level problems on a handful of populations at most. The integration of meta-population and phylogeographical approaches necessitated large-scale sampling of multiple populations at the landscape and regional level, and consideration of the genetic boundaries of related species with overlapping ranges. Enlargement of the ecological canvas has revealed rich patterns of geographical variation including transitions among reproductive systems in zones, such as range margins, where environmental and demographic conditions change (Barrett *et al*. 2001). Studies of geographical variation have provided key insights into the role that ecological conditions play as the principal drivers of evolutionary shifts in reproductive systems.

A particularly striking feature of plant reproductive diversity is that related species often differ in pollination and mating systems, and intraspecific variation in sexual traits is commonplace. This variation provides opportunities to link micro-evolutionary processes to macro-evolutionary patterns. As I discuss in the next section, intra- and interspecific differentiation often represent small-scale versions of patterns characterizing whole lineages. This diversity enables investigation of the ecological and genetic mechanisms driving reproductive character transitions. Phylogenetic and comparative approaches have proven especially useful for reconstructing the evolutionary history of reproductive adaptations (Barrett *et al*. 1996; Weller & Sakai 1999; Case *et al*. 2008), and new methods for investigating ancestral states, correlated evolution and the influence of specific traits on diversification rates are providing exciting new insights into evolution at deeper time scales (Pagel *et al*. 2004; Maddison *et al*. 2007). These advances have benefited from the availability of molecular phylogenies, which form the backbone for reconstructing reproductive character evolution.

This brief historical sketch of major advances in plant reproductive biology has largely focused on the integration of new techniques and approaches. However, I would be remiss in not mentioning several fundamental conceptual advances that have changed the way most plant reproductive biologists view floral function and evolution. First, although controversial when first applied to plants, it is now recognized that sexual selection, including male–male competition and female choice, plays a role in shaping reproductive adaptations even though most flowering plants are hermaphroditic (Willson 1979). Recognition of sexual selection led to the realization that plants may also be subject to Bateman's (1948) principle, namely that male reproductive success is typically limited by mating opportunities, whereas female success is more often limited by resource availability (but see Wilson *et al*. 1994). Second, the appreciation of the seemingly obvious fact that every seed has a mother *and* a father led to the recognition of male function and a plant's paternal role in mating. Although today some workers still persist in equating the relative seed production of hermaphroditic plants with their overall fitness, this is clearly wrong. Total fitness and natural selection on sexual traits can only be estimated properly in hermaphrodites by measuring reproductive success through both female and male function (e.g. Hodgins & Barrett 2008). Recognition of the primary importance of pollen dispersal and outcrossed siring success has resulted in new perspectives on the evolution and function of flowers (Lloyd 1984; Bell 1985; Harder & Barrett 2006) and has helped to integrate work on pollination biology and mating systems, two subfields of reproductive biology that were largely isolated for much of the twentieth century.

## 3. Major evolutionary transitions

**Figure 1.**
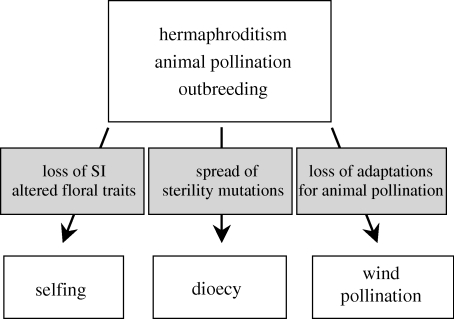
The three major evolutionary transitions in plant reproductive systems discussed in this article—the evolution of selfing from outcrossing, dioecy from hermaphroditism and wind pollination from animal pollination. SI refers to self-incompatibility. Note that in some transitions to selfing and dioecy the immediate ancestors may be wind pollinated.

Evolutionary transitions are functional changes to adaptive traits that spread to replace ancestral conditions because they increase fitness. Transitions take on added significance when they are maintained through multiple speciation events and become characteristic features of lineages. Of particular interest are cases in which replicated character state transitions occur among unrelated lineages because this generally indicates similar selective mechanisms and functional convergence. Today, studies of reproductive transitions in flowering plants are the focus of considerable research in plant evolutionary biology (reviewed in Barrett 2008). This interest arises because transitions affecting modes of reproduction have profound ecological and evolutionary consequences influencing genetic diversity within populations, phenotypic evolution and patterns of diversification. In this section, I review work on three of the most important reproductive transitions in the flowering plants (figure 1).

### (a) The evolution of selfing from outcrossing

The evolution of predominant self-fertilization (autogamy) from high levels of outcrossing is the most frequent reproductive transition in flowering plants (Stebbins 1974). Autogamous species are well represented in many floras, especially those associated with Mediterranean climates, and among successful annual colonizers. There are no accurate estimates of the number of origins of selfing in angiosperms but it is probable that this transition has occurred many hundreds of times. Most transitions to autogamy are likely to go undetected because selfing lineages are often short-lived relative to those composed of outcrossing species. Not surprisingly, given the frequency of this transition and its genetic consequences, it has attracted more attention than any other shift in plant reproductive system (reviewed in Uyenoyama *et al*. 1993).

There are important biological reasons why the evolution of selfing from outcrossing is particularly intriguing to evolutionary biologists. First, the effects of selfing on relative fitness through inbreeding depression are well established; these fitness effects play a major role in determining the dynamics of mating-system evolution (Charlesworth & Charlesworth 1987). Second, the shift to predominant selfing profoundly influences floral evolution, affecting the allocation of resources to floral display, pollen production and aspects of life history (Charnov 1982). Third, selfing individuals can establish colonies at low density, or following long-distance dispersal, and therefore this ability has significant ecological, demographical and biogeographical implications (Baker 1955). Finally, high selfing rates have important genetic and evolutionary consequences influencing population genetic structure (Hamrick & Godt 1996), nucleotide diversity (Wright *et al*. 2008), evolutionary rates (Charlesworth 1992), effective population size (Schoen & Brown 1991) and patterns of evolutionary diversification (Takebayashi & Morrell 2001).

The two most general explanations for why selfing evolves are: (i) the advantage that selfing individuals have over outcrossers when pollinators or mates are scarce (Darwin 1876) and (ii) the genetic transmission advantage through pollen that selfing variants experience because selfers are both the maternal and paternal parents of the seed they produce (Fisher 1941). These two explanations are generally referred to as the ‘reproductive assurance’ and ‘automatic selection’ hypotheses, respectively. There is considerable biogeographical evidence indicating that selfing populations occupy range margins, ecologically marginal sites, or areas with reduced pollinator densities where outcrossers are absent, all circumstances predicted by the reproductive assurance hypothesis. However, surprisingly few field studies have provided experimental evidence in support of the reproductive assurance hypothesis (reviewed in Eckert *et al*. 2006), despite the widespread occurrence of pollen limitation of seed set in animal-pollinated species (Ashman *et al*. 2004). Moreover, even less work has been conducted on the automatic selection hypothesis. Future investigations are needed to determine the relative importance of these two hypotheses in explaining the evolution of selfing in plants.

**Figure 2.**
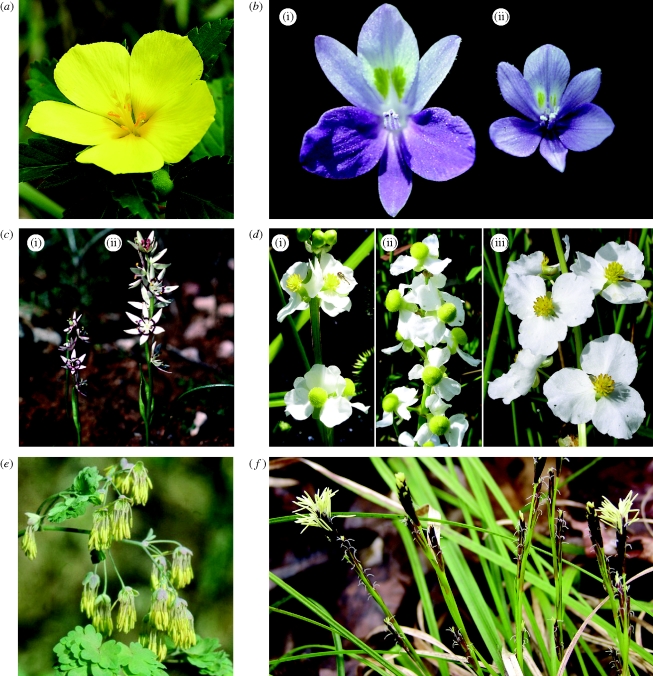
Study systems discussed in the text that have been used for research on plant reproductive diversity. (*a*) A long homostylous flower of *Turnera ulmifolia var. angustifolia* (Turneraceae) from Panama. Plants with this morphology exhibit mixed mating. (*b*) (i) Outcrossing and (ii) selfing flowers of tristylous *Eichhornia paniculata* (Pontederiaceae) from Brazil and Jamaica, respectively. (*c*) (i) Female and (ii) male plants of dioecious *Wurmbea dioica* (Colchicaceae) from South Australia. Note the conspicuous flower size dimorphism, a common feature of dioecious species. (*d*) (i) Hermaphroditic, (ii) female and (iii) male inflorescences of *Sagittaria latifolia* (Alismataceae) from S. Ontario, Canada. The hermaphroditic inflorescence is from a monoecious population and has male flowers at the top of the inflorescence and female flowers at the bottom. The female and male inflorescences are from a dioecious population. (*e*) Inflorescence of a male plant of dioecious wind-pollinated *Thalictrum dioicum* (Ranunculaceae) from S. Ontario, Canada. This genus contains both animal- and wind-pollinated species. (*f*) Inflorescences of monoecious wind-pollinated *Carex pedunculata* (Cyperaceae) from S. Ontario, Canada. Inflorescences are protogynous with male flowers at the top and female flowers below. All photographs taken by the author.

Heterostylous species provide valuable model systems for investigating the transition from outcrossing to selfing. Heterostyly is a floral polymorphism in which populations are composed of two (distyly) or three (tristyly) mating morphs distinguished by the reciprocal arrangements of their sexual organs. This polymorphism promotes pollinator-mediated disassortative (between morph) mating and is maintained in populations by negative frequency-dependent selection. In many heterostylous groups, obligate outcrossing, enforced by heteromorphic self-incompatibility, has been replaced by predominant selfing as a result of the origin of self-compatible homostyles with anthers and stigmas in close contact. The transition from outcrossing to selfing has been detected at the intraspecific level through studies of geographical variation (see below), and by phylogenetic analysis and character mapping in groups with variable mating systems (e.g. Schoen *et al*. 1997). We have studied the evolutionary breakdown of distyly and tristyly in *Turnera* (figure 2*a*) and *Eichhornia* (figure 2*b*), respectively, both neotropical bee-pollinated herbs with widespread geographical distributions (reviewed in Barrett 1989). The genetic mechanisms and evolutionary pathways responsible for the origin of selfing differ between these two genera. In *Turnera*, homostyly arises through recombination between the linked loci governing the style-length/anther-height polymorphism, as has also been well documented in *Primula* (reviewed in Barrett & Shore 2008). In contrast, in *Eichhornia* homostylous selfing forms arise initially through major gene changes to stamen position with subsequent polygenic modifiers reducing flower size (Barrett *et al*. 2009; Vallejo-Marín & Barrett 2009). Hence, in heterostylous species, there is evidence for different genetic and developmental pathways for obtaining the same functional phenotype—plants with the capacity for autonomous self-pollination.

In both *Turnera* and *Eichhornia*, selfing homostyles tend to occupy geographical range margins, a pattern consistent with reproductive assurance. Homostyles in both groups have colonized Caribbean islands, undoubtedly because of the capacity of single individuals to found colonies. There is some evidence in *Turnera* that on large ecologically heterogeneous islands (e.g. Cuba and Jamaica), the re-establishment of outcrossing in homostyles has occurred through selection on quantitative genetic variation governing stigma–anther separation (figure 2*a*). This suggests that the acquisition of selfing is not necessarily always a ‘one-way street’ as Stebbins (1974) originally proposed.

The question of whether selfing lineages often persist and contribute to phyletic evolution is unresolved (Takebayashi & Morrell 2001). A key issue in determining whether selfing is an evolutionary dead end concerns the degree of selfing considered. Highly selfing species (e.g. *s* = >0.90) usually possess the selfing syndrome (e.g. very small flowers, reduced pollen production, low nectar production) and it seems unlikely that populations with this mating system could re-evolve high levels of outcrossing and contribute to adaptive radiation. On the other hand, in selfing species with moderate rates of outcrossing (e.g. *t* = 0.25), a return to higher levels may be possible if sufficient genetic variation for floral traits affecting pollinator visitation is present within populations. Homostylous populations of *Turnera* in the Caribbean display a wide range of selfing rates but those with very small flowers, such as occur on some Bahamian islands (see Barrett 1989, figure 2*d*), may have gone past the point of no return. Determining if there is a threshold selfing rate beyond which the evolution of the selfing syndrome is inevitable would be valuable, and more generally work on the potential nonlinear relations between mating patterns, genetic diversity and selection response in plant populations is needed.

Homostyles in *Eichhornia*, like *Turnera*, have arisen on multiple occasions and exhibit geographically marginal distributions. We have focused most of our attention on *E. paniculata* because this species displays one of the widest ranges of outcrossing rate reported in flowering plants. Populations in northeast Brazil are large flowered, primarily tristylous and outcrossing, whereas small-flowered autogamous populations occur on Jamaica, Cuba and in scattered localities in Nicaragua and Mexico; populations with mixed mating and intermediate flower sizes connect these extremes. Studies on the inheritance of mating-system modification, and comparisons of the genetic relationships of populations using nuclear DNA sequences, have provided evidence for multiple independent transitions to predominant selfing (Barrett *et al*. 2009). *Eichhornia paniculata* is an annual of ephemeral ponds and populations commonly experience dramatic fluctuations in size and frequent colonization–extinction cycles as well as frequent dispersal events (Husband & Barrett 1998). Our work has shown that the joint action of stochastic forces and natural selection can destabilize tristyly causing the loss of morphs from populations and the subsequent spread of self-pollinating variants through automatic selection and reproductive assurance (Barrett *et al*. 1989). The shift to selfing is an example of a transition in mating pattern triggered by genetic drift and fulfils some of the key conditions identified in Sewall Wright's shifting balance theory of evolutionary change (Coyne *et al*. 1997). Our current work is examining the genomic consequences of replicated transitions from outcrossing to selfing in *E. paniculata* to determine if general patterns are evident.

### (b) The evolution of dioecy from hermaphroditism

The evolution of populations with females and males (dioecy) from hermaphroditism has interested evolutionary biologists since Darwin (1877) struggled to understand the circumstances that might favour the unisexual condition in angiosperm species. In immobile organisms, like plants, it is not immediately obvious why hermaphroditism should be abandoned in favour of unisexuality, because a reduction or loss of pollen vectors would severely compromise individual fitness. Another difficulty for the evolution of dioecy is that genetic transmission is halved for unisexuals in comparison with hermaphrodites, which can acquire fitness through either of two sexual avenues. Finally, for dioecy to evolve from hermaphroditism requires the spread of sterility mutations that under most circumstances would be deleterious to fitness. These hurdles present an intriguing puzzle for evolutionary biologists.

Dioecy is not especially common in flowering plants, occurring in approximately 6–7% of species; however, it is represented in close to half of all angiosperm families and has evolved on at least 100 occasions (Renner & Ricklefs 1995; Charlesworth 2002). The two main evolutionary routes by which dioecy evolves are referred to as the ‘gynodioecy pathway’, in which populations with females and hermaphrodites (gynodioecy) are an intermediate condition, and the ‘monoecy pathway’, in which selection on quantitative variation in sex allocation in monoecious ancestral populations is thought to be involved. Monoecy is the condition in which individual plants possess both female and male flowers. Disruptive selection on genetic variation in floral sex ratios could in principle result in female and male specialists. We know quite a bit about the gynodioecy pathway but curiously, given that monoecy is very commonly associated with dioecy at the generic level (Renner & Ricklefs 1995), the pathway from monoecy to dioecy has largely been neglected. Our recent work on *Sagittaria*, showing that dioecy may evolve from monoecy via the gynodioecy pathway as a result of the spread of females in monoecious populations (Dorken & Barrett 2004), indicates that in some groups the two pathways to dioecy may not be as distinct as is generally assumed and that an ancestral condition of monoecy does not guarantee the evolution of dioecy via the monoecy pathway.

Dioecy is commonly associated with a suite of life history and reproductive traits. For example, unlike predominant selfing, dioecy occurs most commonly in long-lived species and is rare in annuals. Understanding the functional basis of these associations can provide useful clues about the ecological factors causing transitions to unisexuality. Using a molecular phylogeny of the angiosperms, and maximum likelihood approaches that take into account the phylogenetic non-independence of species, Vamosi *et al*. (2003) found that dioecy was associated with several traits including the woody growth form, abiotic pollination, small inconspicuous white or green flowers and fleshy fruits. Why do these trait associations occur? Unfortunately, there are no clear answers to this question. This is because it has not been possible to determine with any confidence the order of acquisition of these traits in relation to the origins of dioecy. For example, is dioecy more likely to evolve in fleshy-fruited lineages, or does dioecy favour the evolution of fleshy fruits? These questions are difficult to answer because the presence of fleshy fruits is also correlated with woodiness, another correlate of dioecy. The intercorrelation of traits makes it tricky to tease apart the potential mechanisms involved in the evolution of dioecy. Future progress is more likely to come from analysing smaller-scale phylogenies of families or genera polymorphic for sexual systems and the traits of interest. However, finding groups that meet these criteria and have multiple independent transitions to dioecy will be challenging.

A recent study of the evolutionary history of sexual systems in *Wurmbea* (figure 2*c*; Case *et al*. 2008), a genus of geophytes (plants with bulbs or corms) native to Southern Africa and Australia, provides a salutary warning that reconstructing evolutionary history is no easy task, despite the profusion of trees with characters mapped onto them in the current literature. In this study, the largest source of uncertainty was the interspersion of hermaphroditic and sexually dimorphic taxa across phylogenetic trees owing to the evolutionary lability of sexual systems in the genus. This diversity includes both the origin of dioecy and its reversion to hermaphroditism. Such variation complicates inferences on ancestral nodes and the determination of the statistical confidence of character state transitions. A particularly thorny issue came to light from sampling multiple populations of *W. dioica* and *W. biglandulosa*, two species that have been the main focus of micro-evolutionary work on the ecological mechanisms driving the evolution of dioecy in *Wurmbea* (reviewed in Barrett & Case 2006). The interest in these species arises because of their widespread distributions and the fact that they are both polymorphic for sexual-system variation with both hermaphroditic and gender dimorphic populations. Unexpectedly, and despite a recent taxonomic treatment of the group, both species were found to be non-monophyletic. Clearly, this situation makes the interpretation of character evolution uncertain. The problems encountered in this study are unlikely to be restricted to *Wurmbea*; geographically widespread taxa are commonly used to investigate the ecological mechanisms driving transitions specifically because they display ‘intraspecific variation’. Investigators interested in character state transitions should sample widely to confirm species boundaries and the monophyly of species under investigation. Unfortunately, this is often not done in phylogenetic studies, especially in large groups where extensive taxon sampling is necessary.

The most widely accepted hypothesis for the function of dioecy is that it is a mechanism of inbreeding avoidance preventing self-fertilization. Gender dimorphism commonly evolves from self-compatible rather than self-incompatible ancestors, a pattern consistent with the anti-selfing hypothesis. Theoretical models identify three main sets of factors that are important when considering the evolution of dioecy: selfing and inbreeding depression, the optimal allocation of resources to female and male fertility and the genetic control of sex determination (Charlesworth 1999). Of primary significance is to determine the relations between rates of selfing and inbreeding depression in ancestral hermaphrodite populations as these will influence whether unisexual variants can spread. There is a considerable amount of theoretical work on the values that these parameters can take under both nuclear and cytoplasmic inheritance of sex determination. However, much less is known about the biological factors responsible for increases in selfing rate in ancestral hermaphrodite populations and our understanding of the ecological context in which dioecy evolves lags well behind the theory. For example, stressful environmental conditions have often been linked to the evolution of unisexuality (Darwin 1877, p. 301; reviewed in Ashman 2006). Limited resources under harsh conditions may lower the seed fertility of hermaphrodites, and/or stress could intensify the magnitude of inbreeding depression. Both factors should give an advantage to unisexuals but as yet definitive ecological studies have not been undertaken to assess their relative importance.

Most herbaceous species that are dioecious are perennial and many form extensive clones. This raises the possibility that geitonogamous selfing may play a role in favouring unisexuality. We are investigating whether clonality influences the origin and maintenance of dioecy in *Sagittaria latifolia*, a native of North American wetland habitats that possesses both hermaphroditic (monoecious) and dioecious populations (figure 2*d*). Clonal growth results in restricted foraging of pollinators (flies and small bees) and opportunities for considerable geitonogamous selfing in monoecious populations. If inbreeding depression is strong, this should favour the spread of unisexual variants, as they would benefit from outbreeding advantage. We have demonstrated that selfing rates and levels of inbreeding depression in monoecious populations can exceed theoretical values predicted to favour the spread of unisexual variants. This provides a plausible mechanism to explain how dioecy may have become established in this species (Dorken *et al*. 2002). Clone sizes in *S. latifolia* are generally larger in dioecious than in monoecious populations, a pattern also consistent with the proposed ecological mechanism. However, our evidence is not definitive because we cannot rule out the alternative scenario; large clone size may have evolved after the establishment of dioecy, because the mating costs imposed by geitonogamy in monoecious populations are removed in unisexual plants. Knowledge of the evolutionary history of dioecy and clone size in *Sagittaria* would be useful for distinguishing between these alternative hypotheses. Unfortunately, dioecy is restricted to a single species in this genus preventing any meaningful analysis of evolutionary history. Comparative studies of the origins of dioecy in plant groups containing multiple dioecious and hermaphroditic species that vary in clone size (e.g. grasses, sedges and seagrasses) could be valuable for evaluating the idea that geitonogamous selfing plays a role in the evolution of unisexuality in plants.

### (c) The evolution of wind pollination from animal pollination

In comparison with the two preceding reproductive transitions, our understanding of the evolutionary mechanisms responsible for the origin of wind pollination (anemophily) is rudimentary at best. There is virtually no theory development in this area, nor empirical work on the micro-evolutionary forces responsible for the selection of wind pollination. This gap in our understanding is surprising because this transition represents one of the most profound transformations in the reproductive biology of angiosperms and affects many aspects of floral evolution, ecology and genetics. Approximately 10 per cent of angiosperm species rely on wind pollination and are represented in most ecosystems, dominating in some (e.g. grasslands, salt marshes). Wind pollination is known to have evolved at least 65 times from animal-pollinated ancestors (Linder 1998) and is not the primitive condition in angiosperms that was originally assumed. Nevertheless, in comparison with animal pollination, wind pollination is often described as a random and wasteful process involving a huge loss of male gametes during pollen dispersal because of the vagaries of changing atmospheric conditions.

The characterization of anemophily as an inefficient pollination system presents evolutionary biologists with a conundrum—if wind pollination is so inefficient, why has it evolved repeatedly from animal pollination? The most common answer to this question is that wind pollination is likely to evolve when ecological conditions render animals less reliable as vectors for pollen transfer. This implies that in populations receiving unsatisfactory pollinator service, seed set is pollen limited and wind pollination evolves because it provides reproductive assurance in much the same way as self-pollination relieves pollen limitation. Although this seems entirely plausible, there is remarkably little concrete evidence to support this hypothesis; moreover, as discussed earlier, the loss of pollinator service is also the principle mechanism explaining the evolution of selfing from outcrossing. How could the same requirement—reproductive assurance—result in such widely different reproductive outcomes? We have addressed this issue using two complementary approaches. The first uses comparative analyses to examine if the traits in ancestral populations might influence whether wind pollination is likely to evolve to achieve reproductive assurance. The second involves field experiments to determine if the assumption of pollination inefficiency is indeed valid for wind-pollinated species.

We conducted a large-scale comparative analysis of angiosperms and found that wind pollination evolves more often in animal-pollinated lineages that possess unisexual flowers as a result of either monoecy or dioecy (Friedman & Barrett 2008). In these lineages, autonomous intraflower self-pollination would be prevented because perfect (hermaphroditic) flowers are required for this form of selfing to occur. Thus, wind pollination may replace selfing as a mechanism providing reproductive assurance in these groups. According to this hypothesis, insufficient pollinator service resulting in pollen limitation could elicit two quite different evolutionary transitions, depending on the floral condition of ancestral populations. In populations with hermaphroditic flowers, autonomous self-pollination would relieve pollen limitation resulting in the evolution of selfing. This could be easily achieved through reductions in stigma–anther separation and flower size. In contrast, in populations with unisexual flowers, such changes would be mechanically precluded and wind pollination may serve the same role by increasing the proficiency of cross-pollen dispersal. This proposition emphasizes the importance of phyletic heritage in determining evolutionary trajectories and suggests that solutions to pollen limitation other than autonomous selfing can evolve and maintain outcrossing (see Harder & Aizen 2010).

Our study evaluating pollination efficiency in wind-pollinated plants involved measuring the amount of pollen produced and captured by stigmas in 19 wind-pollinated herbaceous species (Friedman & Barrett 2009*a*). Although the range in the proportion of pollen produced that was captured (mean = 0.32%, range 0.01–1.19%) was somewhat lower than values for animal-pollinated species (0.03–1.9%; Harder 2000), our data do not support the prevailing view that pollen dispersal in wind-pollinated plants is much more inefficient than in animal-pollinated plants. Both pollination systems are characterized by large transport losses. Interestingly, in only one of 10 wind-pollinated species we examined was there evidence of pollen limitation of seed set. Future studies are needed on a diversity of wind-pollinated taxa to assess the frequency of pollen limitation in comparison with animal-pollinated plants, where it appears to be surprisingly common (Ashman *et al*. 2004). If reproductive assurance turns out to be the chief reason that wind pollination evolves in the flowering plants, pollen limitation should be less common than in animal-pollinated species. This idea could be tested experimentally through comparative studies.

Our understanding of the origins of wind pollination has been limited by the striking paucity of plant groups in which there are clear evolutionary transitions from animal to wind pollination (e.g. *Thalictrum*, figure 2*e*). This contrasts with the preceding examples of transitions to selfing and dioecy where interspecific, and especially intraspecific, variation has been profitably exploited to understand evolutionary mechanisms. To my knowledge, there is not a single convincing example of intraspecific differentiation in pollination systems in which a species maintains separate populations adapted to animal pollination versus wind pollination. There is also a general absence of basic information about the reproductive biology of anemophilous species and it seems probable that some generalizations developed for animal pollination may require revision when applied to wind-pollinated species (discussed more fully in Friedman & Barrett 2009*a*). For example, many anemophilous plants possess unisexual flowers and are protogynous (female function before male function); traits usually considered as anti-selfing mechanisms in animal-pollinated species. We recently investigated mating in seven monoecious wind-pollinated *Carex* species (figure 2*f*) possessing these traits and unexpectedly discovered that they all appear to be largely selfing as a result of geitonogamy (Friedman & Barrett 2009*b*). Future functional studies linking pollen dispersal and mating through the use of genetic markers have the potential to reveal complex and fascinating new details on this most neglected of pollination systems.

## 4. Future developments

In the preceding discussion, I identified particular challenges and gaps in our understanding of several areas concerned with the evolution of plant reproductive diversity. I conclude by briefly considering what lies ahead and how the field of plant reproductive biology is likely to develop over the next few decades.

Genomics is changing many areas of inquiry and plant reproductive biology is no exception. Although in its infancy, the application of molecular population genetic approaches will surely provide important insights into the mechanisms governing changes in reproductive system as well as their genomic consequences (Wright *et al*. 2008). For example, molecular genetic approaches can inform our understanding of how, when and how many times particular transitions have evolved, as has recently been done for the evolution of selfing in *Arabidopsis* and *Capsella*, respectively (Tang *et al*. 2007; Foxe *et al*. 2009). To understand the proximate mechanisms governing pollination and mating, it is also crucial that we identify the genes responsible for key reproductive traits. A start has been made through work on the genes controlling self-incompatibility in diverse families (Franklin-Tong 2008), andromonoecy in melons (Boualem *et al*. 2008) and floral traits in wind- versus animal-pollinated species of *Thalictrum* (Di Stilio *et al*. 2009). It will be fascinating to discover in cases involving the three reproductive transitions that I have considered here, how often homologous genes are involved given that each transition has occurred on numerous occasions among angiosperm families. There is much to learn about the molecular and developmental basis of plant reproductive diversity.

Molecular and developmental studies can tell us how sexual diversity may arise but they cannot tell us why. Field studies of the ecological genetics of natural populations are required to determine the selective mechanisms responsible for the evolution and maintenance of reproductive traits. We now have the analytical tools to measure natural selection in wild populations, estimate the heritability and evolvability of reproductive traits using quantitative genetic approaches and, by using microsatellite markers, determine mating success through male function. Combining these approaches with imaginative field experiments involving manipulation of the abiotic and biotic features of populations and of the floral phenotypes themselves should help in isolating the ecological drivers of changes in mating strategy. Fortunately, the sedentary nature of plants and the fact that they can be easily cultured, crossed and cloned makes them ideal organisms for manipulative field experiments. Lastly, global environmental change has and will have manifold effects on the reproductive biology of plant populations (e.g. Barrett 2000; Aguilar *et al*. 2006; Aizen & Vázquez 2006). Climate change, habitat fragmentation and the spread of invasive species will all directly impact the capacity of plant populations to reproduce successfully, with consequences for their demography, evolution and long-term persistence. Further understanding of the biology of plant reproduction will therefore be of crucial importance for dealing with these environmental challenges and for maintaining biodiversity, genetic resources and human well-being.
